# 5,8-Quinolinedione Scaffold as a Promising Moiety of Bioactive Agents

**DOI:** 10.3390/molecules24224115

**Published:** 2019-11-14

**Authors:** Monika Kadela-Tomanek, Ewa Bębenek, Elwira Chrobak, Stanisław Boryczka

**Affiliations:** Department of Organic Chemistry, Faculty of Pharmaceutical Sciences in Sosnowiec, Medical University of Silesia, 4 Jagiellońska Str., 41-200 Sosnowiec, Poland; ebebenek@sum.edu.pl (E.B.); echrobak@sum.edu.pl (E.C.); boryczka@sum.edu.pl (S.B.)

**Keywords:** 5,8-quinolinedione, streptonigrin, biological activity, NAD[P]H-quinone oxidoreductase

## Abstract

Natural 5,8-quinolinedione antibiotics exhibit a broad spectrum of activities including anticancer, antibacterial, antifungal, and antimalarial activities. The structure–activity research showed that the 5,8-quinolinedione scaffold is responsible for its biological effect. The subject of this review report is a presentation of the pharmacological activity of synthetic 5,8-quinolinedione compounds containing different groups at C-6 and/or C-7 positions. The relationship between the activity and the mechanism of action is included if these data have been included in the original literature. The review mostly covers the period between 2000 and 2019. Previously published literature data were used to present historical points.

## 1. Introduction

Natural products are an important source of medicinal substances used in the therapy of various ailments including infectious, cardiovascular, neurological, and oncological diseases. Such substances are produced by plants, microorganisms, and invertebrates. About half of the currently used antibiotics come from the strain of *Streptomyces* bacteria, a microorganism which is widespread in nature [1].

**Figure 1:**
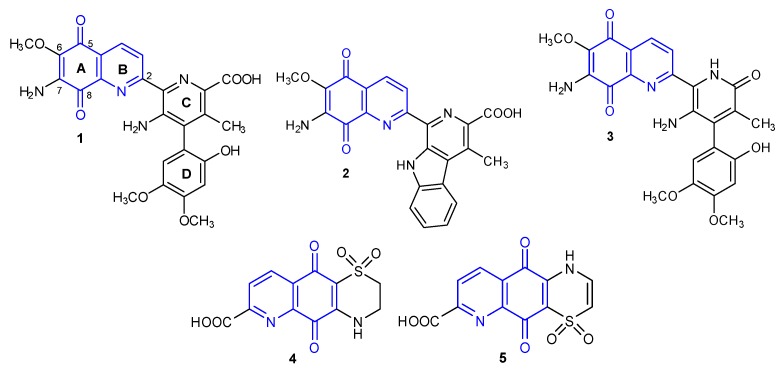
Structure of natural 5,8-quinolinedione antibiotics **1**–**5**.

In 1959, the bioactive 7-aminoquinone called Streptonigrin **1** (Figure 1) was isolated from *Streptomyces flocculus* [2]. This alkaloid was also found in *Actinomyces albus var bruneomycini*, *Streptomyces rufochromogenes,* and *Streptomyces echinatus* [3,4].

Based on chemical degradative methods and spectral analysis, Rao et al. developed the chemical structure of Streptonigrin **1** [5]. Finally, the structure of compound **1** was confirmed by x-ray crystallography in 1975 [6]. The total synthesis of the alkaloid **1** has been carried out by two research groups (Weinreb et al. and Kende et al.) from 1980 to 1982 [7,8].

In the following years, Lavendamycin **2** [9,10] and Streptonigron **3** were isolated from the *Streptomyces* species (Figure 1) [9,11]. Another two compounds of 5,8-quinolinedione named Ascidiathiazones A **4** and B **5** were obtained from a New Zealand ascidian *Aplidium* species in 2005 (Figure 1) [12,13]. Naturally occurring 5,8-quinolinediones **1–5** exhibit a wide spectrum of biological properties including anticancer, antimicrobial, antiviral, antimalarial, and anti-inflammatory activities [9,12,14,15,16,17,18,19,20,21,22]. The most attractive anticancer activity is shown by compound **1**, which has been tested as an anti-leukemia drug. However, a high degree of toxicity caused the termination of this research at phase II of clinical trials [9].

The multidirectional biological activity of natural compounds **1**–**5** results from their structural diversity and the possibility of interaction with various molecular targets. The mechanism of biological action of the compounds containing the 5,8-quinolinedione moiety largely depends on their ability to form radicals in vivo. Numerous studies have confirmed that nicotinamide adenosine diphosphate (NADP) or NADPH-dependent quinone oxidoreductase (NQO1) are important molecular targets for new derivatives based on the structure of 5,8-quinolinedione [18,20,23].

Research into the structure–activity relationship for natural antibiotics **1**–**5** has shown that the 5,8-quinolinedione scaffold is essential for ensuring biological activity [9,18,24,25]. Introduction of various groups at the C-6 and C-7 positions significantly affects the biological properties of the compounds. In many cases, modification of the 5,8-quinolinedione moiety at the C-2 position reduces activity when compared to compounds not substituted at this position [26,27,28].

Most synthetic compounds containing the 5,8-quinolinedione scaffold exhibit better activity and lower toxicity than natural antibiotics **1**–**3** [17,29,30]. This review presents selected examples of synthetic derivatives of 5,8-quinolinedione and their biological activity.

## 2. Synthesis of 5,8-Quinolinedione Compounds

**Scheme 1:**
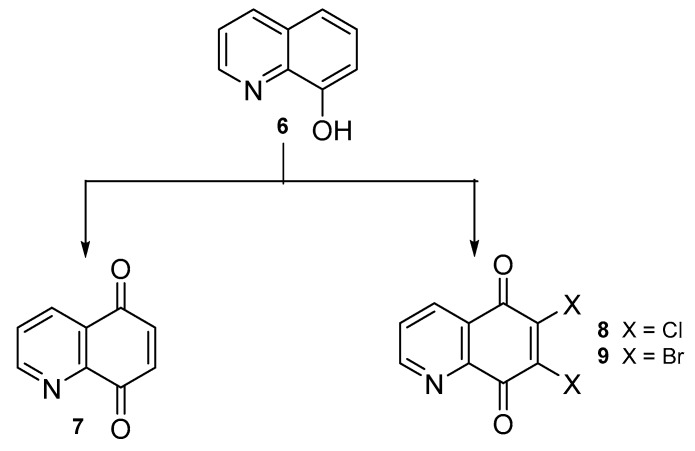
Synthesis of compounds **7**–**9**.

Synthetic derivatives of 5,8-quinolinedione are commonly obtained from 5,8-quinolinedione **7** or 6,7-dihalogen-5,8-quinolinediones **8**–**9**. The synthetic pathways of compounds **8**–**9** from 8-hydroxyquinoline **6** are depicted in Scheme 1 and reaction conditions are presented in Table 1.

**Scheme 2:**
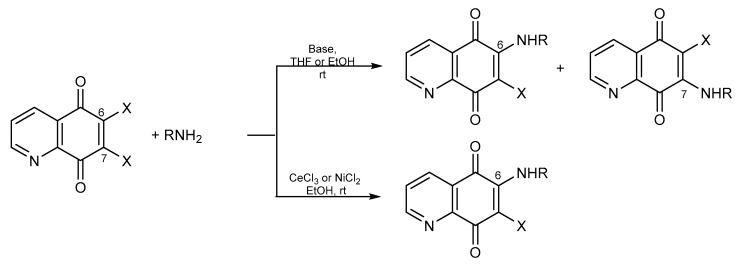
Synthesis of amine derivatives of 5,8-quinolinedione.

**Scheme 3:**
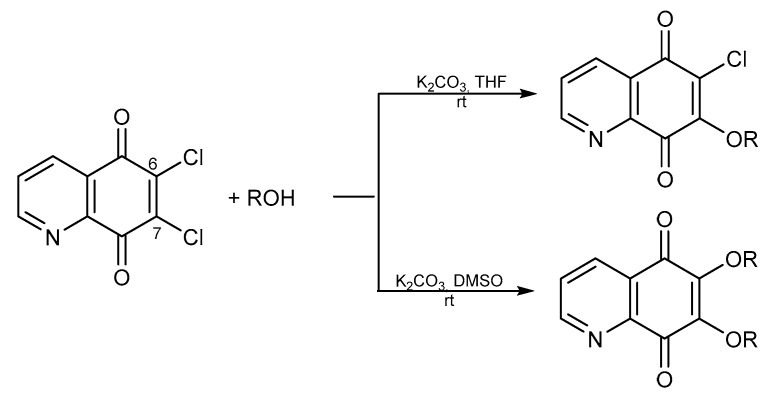
Synthesis of alkoxy derivatives of 5,8-quinolinedione.

**Scheme 4:**
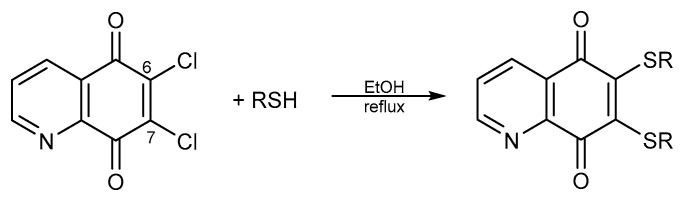
Synthesis of thiol derivatives of 5,8-quinolinedione.

Treatment of compounds **7**–**9** with different nucleophilic agents like amines, alcohols, or thiols, led to the formation of mono- or disubstituted derivatives (Scheme 2, Scheme 3 and Scheme 4).

Reaction of 5,8-quinolinediones **7**–**9** with an amine under basic conditions led to a mixture of 6- or 7-mono-substituted compounds, but the ratio of the products depended on the type of solvent. Use of an aprotic solvent such as tetrahydrofuran (THF) or dimethylformamide (DMF) led to the 7-amino compound as a major product. The 6-substituted compounds were formed in a protic solvent like water or ethanol (Scheme 2) [40,45,46,47,48]. Moreover, 6-amino-5,8-quinolinedione could be obtained selectively in reaction with the addition of a catalyst like CeCl_3_ or NiCl_2_ in the presence of ethanol (Scheme 2) [24,26,49,50]. Modification of 6-amino-5,8-quinolinedione derivatives resulted in 6-amino-7-thiol-5,8-quinolinedione and multicyclic compounds [24,26,38].

The alkoxy derivatives were obtained in the reaction of 6,7-dichloro-5,8-quinolinedione **8** with alcohol in the presence of potassium carbonate. However, if the reaction was conducted in THF or dimethyl sulfoxide (DMSO) as a solvent, then 7-mono or 6,7-disubstituted products were obtained, respectively (Scheme 3) [28,51,52].

Treatment of compound **8** with arylthiols in the presence of ethanol resulted in 6,7-diarylthiol derivatives (Scheme 4) [53].

## 3. Biological Activity

### 3.1. Anticancer Activity

The World Health Organization (WHO) reports that cancer is the first or second main cause of death in 71 of 127 countries. The International Agency for Research on Cancer (IARC) estimated that only in 2018, 18.1 million new cases were diagnosed and 9.6 million people died of cancer. According to the GLOBOCAN 2018 research, the most common types of cancers are lung and breast cancer, which accounted for over 23% of new oncological cases [54].

Several compounds containing the 5,8-quinolinedione moiety that exhibit anticancer activity have been described in the literature. Ling et al. obtained a series of 6- and 7-arylamino-5,8-quinolinediones that were tested against drug sensitive (HeLeS3) and multidrug resistant (KB-vin) cell lines [37].

**Figure 2:**
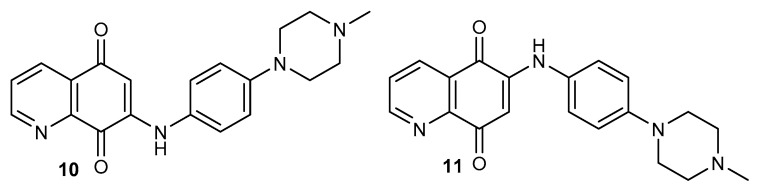
Structure of [4-(4-methylpiperazin-1-yl)phenyl]aminoquinoline-5,8-diones **10**–**11**.

Compounds **10**–**11** (Figure 2) were characterized by high anticancer activity against both cell lines and their IC_50_ values were within the range of 0.59–1.52 µM. The 7-substituted derivative **10** exhibited 1.4-time and 1.6-time higher activity than compound **11** against HeLeS3 and KB-vin, respectively. Further research has shown that both compounds cause an NQO1-dependent antiproliferative effect and induce a dose-dependent lethal mitochondrial dysfunction in the tested cell lines. Derivative **10** exhibited anticancer activity, but did not affect a normal cell line. Moreover, it induced apoptosis by upregulating Bcl-2 protein and reducing Bax protein and cleaved caspase-3 [37].

A small library of arylamine derivatives of 5,8-quinolinedione were prepared by Ryu et al. [50,55,56].

**Figure 3:**
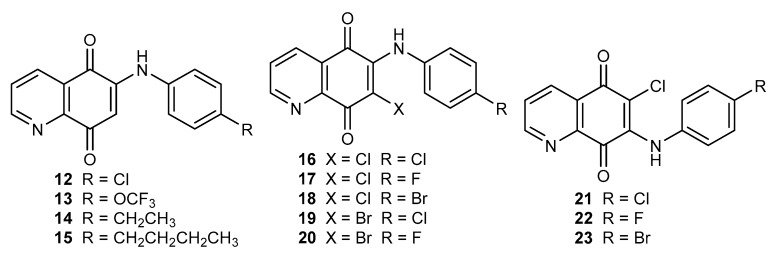
Structure of arylamino-5,8-quinolinediones **12–23**.

Derivatives **12**–**23** (Figure 3) were tested against lung cancer (A549), ovary adenocarcinoma (SK-OV-3), and malignant melanoma (SK-MEL-2) cell lines. In the series of **12**–**15**, compound **13** exhibited high activity against all tested cell lines with the IC_50_ range of 0.36–0.85 µg/mL. Moreover, this derivative had a higher activity than cisplatin. Compound **12** demonstrated a selective activity against the A549 cell line, which was 2- and 3-times higher than Streptonigrin **1** and cisplatin, respectively. Additionally, for compounds **12**–**15**, the effect on NQO1 activity was evaluated using the cytosolic fractions of the A549 cell line. This research showed that the type of substituent affects the NQO1 activity as follows: propyl (**15**) > chloride (**12**) > ethyl (**14**) > trifluorometoxy (**13**) [50]. In order to check the influence of the substituent at the C-7 position, 6-arylamino-7-halogen-5,8-quinolinediones **16**–**20** were obtained (Figure 3) [55]. Compounds **12**, **16**, and **19** differed only in the C-7 position substituent. Comparing their anticancer activity against the A549, SK-OV-3, and SK-MEL-2 cell lines, it showed that the introduction of a halogen atom at the C-7 position causes a decrease in activity [50,55]. However, the derivatives containing a chlorine atom (**16**–**17**) at this position exhibited higher activity than those containing a bromine atom (**19**–**20**). The structure–activity relationship showed that the R group affects the anticancer activity, but there was no correlation between the properties of the R group and the activity against the tested cell lines [55].

Natural 5,8-quinolinedione antibiotics contain an amine group at the C-7 position; for this reason, Ryu et al. obtained 7-arylamine-6-chloro-5,8-quinolinedione **21**–**23** (Figure 3) [56]. The study showed that compounds **16**–**18** and **21**–**23** had a similar activity against the SK-OV-3 and SK-MEL-2 cell lines. The 7-arylamine-6-chloro-5,8-quinolinedines **21**–**23** was characterized by a lower activity against lung cancer (A549) than derivatives **16**–**18** (Figure 3) [55,56]. In the series of 7-arylamine compounds **21**–**23**, the lowest IC_50_ value against the A549 cell line was exhibited by 6-chloro-7-(4-fluorphenylamine)-5,8-quinolinedione **22**, having activity comparable with that of cisplatin (IC_50_ = 1.80 µg/mL) [56].

**Figure 4:**
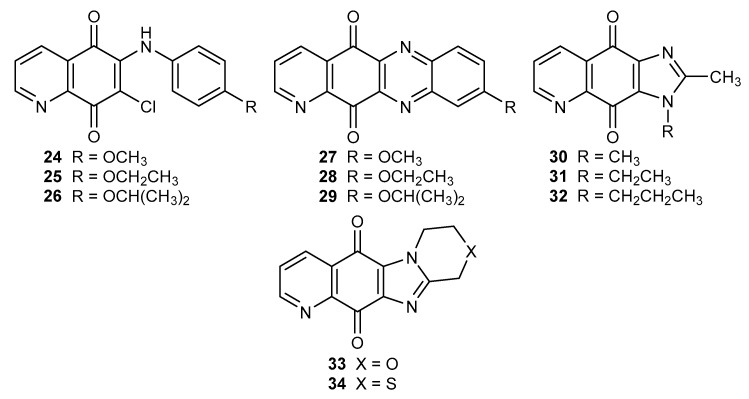
Structure of 6-arylamine **24**–**26** and multicyclic compounds of 5,8-quinoliniedione **27–34**.

The 6-arylamine derivatives **24**–**26** were used as substrates for the synthesis of tetracyclic compounds **27**–**29** (Figure 4). Both series of compounds were tested against ovary adenocarcinoma (SK-OV-3), malignant melanoma (SK-MEL-2), lung (A549), brain cancer (XF 498), and colon (HCT 15) cancer cell lines [24].

A comparison of the activity of derivatives **24**–**25** and **27**–**28** showed that tetracyclic compounds **27**–**28** exhibited a higher anticancer activity in relation to all tested cell lines. The IC_50_ value for **29** was in the range of 0.12–0.21 µM, and was comparable with the activity of doxorubicin against the SK-OV-3 and XF 498 cell lines [24].

Suh et al. described tri- and tetracyclic (**30**–**32** and **33**–**34**, respectively) (Figure 4) imidazo-5,8-quinolinedione analogues, which exhibited potential anticancer activity against the A549, SK-OV-3, SK-MEL-2, XF 498, and HCT 15 cell lines. A comparison of the IC_50_ values of **30**–**32** derivatives against the tested cell lines suggested that side chain elongation of the substituent led to a decrease in activity. Compounds **33** and **34** showed a similar anticancer effect against the tested cell lines. In the case of the SK-OV-3 line, compound **33** exhibited an activity 3.5-times higher than **34** [21].

**Figure 5:**
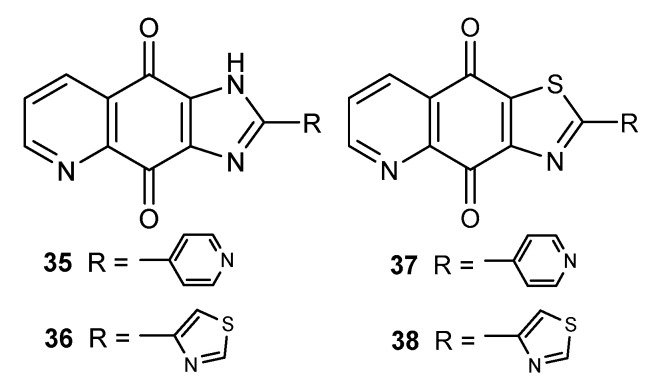
Structure of imidazo-5,8-quinolindiones **35**–**36** and thiazolo-5,8-quinolinediones **37**–**38**.

The indoleamine 2,3-dioxygenase 1 (IDO1) enzyme catalyzes degradation of tryptophan to N-formyl-kynurenine. This reaction initiates the kynurenine pathway leading to the synthesis of nicotinamide adenine dinucleotide (NAD), which is used to transfer electrons in many enzymatic reactions. Many studies have confirmed that IDO1 has an influence on the development of many types of human tumors [57,58,59]. One of the group that exhibited promising IDO1 inhibitory activity was imidazo-5,8-quinolindione compounds **35**–**36** and thiazolo-5,8-quinolinediones **37**–**38** (Figure 5) [38].

The inhibitory rates of compounds **35**–**36** and **37**–**38** at 1 µM were 65.5–76.0%. However, derivatives containing 4-thiazolyl substituent as the R group (**36** and **38**) showed a higher activity against IDO1 than epacadostat (INCB24360) used as the positive control. The IC_50_ values determined for **36** and **38** were equal to 61 nM and 18 nM, respectively. The cytotoxic activity was evaluated against human breast (MCF-7) and cervical (HeLa) cancer cell lines. The in vivo test showed that **38** reduced kynurenine levels in plasma by 30%. The CC_50_ values were significantly higher than the IC_50_ values, meaning that the tested compounds were not cytotoxic in their effective concentration against IDO1 [38].

Enzymes controlling the cell cycle process include phosphatase Cdc25, which exists as three homologues denoted as Cdc24A, Cdc25B, and Cdc25c. Overexpression of Cdc25B and Cdc25c has been observed in many human tumors and is considered to be oncogenic [60]. Lazo et al. examined 5,8-quinolinedione compounds as potential inhibitors of recombinant human Cdc25B_2_ [47].

**Figure 6:**
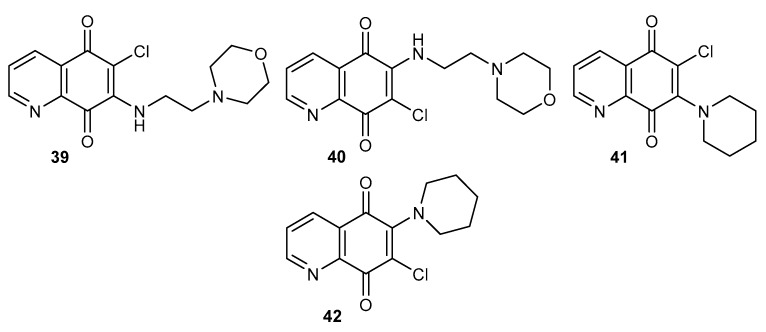
Structure of alkylamino-5,8-quinolinediones **39**–**42**.

In the series of tested compounds, the highest inhibitory activity was shown by compounds containing the 2-morpholin-4-ylethylamine substituent, **39** and **40** (Figure 6). A comparison of the activity of these two compounds showed that the position of amine substituent affects the activity. The IC_50_ value for **39** was 3-times lower than that calculated for **40**, and they were equal to 0.21 µM and 0.82 µM, respectively [47]. The in vitro tests against breast cancer cells showed that derivative **39** selectively and irreversibly inhibits Cdc25 phosphatases, which results in a decrease in the Cdc2 kinase level and causes the arrest of the cells in the G1 and G2/M phases of the cell cycle [47,61].

Compounds **41**–**42** (Figure 6) were designed as inhibitors of Cdc2 and ERK proteins [62]. Extracellular signal-regulated kinase (ERK) plays an important role in the signal pathway, which controls cancer cell proliferation and transformation [63]. The preliminary test showed that only compound **42** induces cytotoxicity in the lung cancer cell line (A549) significantly, and the IC_50_ value was estimated at 5 µM. Hsu et al. found that **42** inhibited the phosphorylated sites of Cdc2 and increased the G_2_/M arrest in lung cancer cells. The blockade of ERK by **42** may result in the inhibition of proliferation and apoptosis of A549 cells [62].

**Figure 7:**
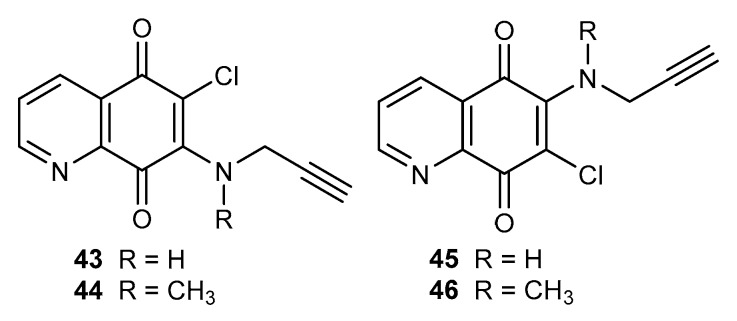
Structure of alkynylamino-5,8-quinolinediones **43**–**46**.

The alkyne moiety, which is one of the most important groups in medical chemistry, affects the biological and chemical properties of compounds that contain this fragment [64,65,66]. For this reason, acetylenic amine derivatives of 5,8-quinolinedione **43**–**46** (Figure 7) were prepared. The antiproliferative activity was tested against melanoma (C-32), glioblastoma (SNB-19), and breast cancer (T47D) cell lines [48].

Derivatives **43**–**44** and **45**–**46** exhibited a higher efficiency against the tested cell lines than cisplatin. In both groups of compounds, it was observed that an introduction of the methyl group led to a decrease in the activity. A comparison of the activity of 6- and 7-amino substituted derivatives showed that **43**–**44** had a lower anticancer efficiency than **45**–**46** [48].

**Figure 8:**
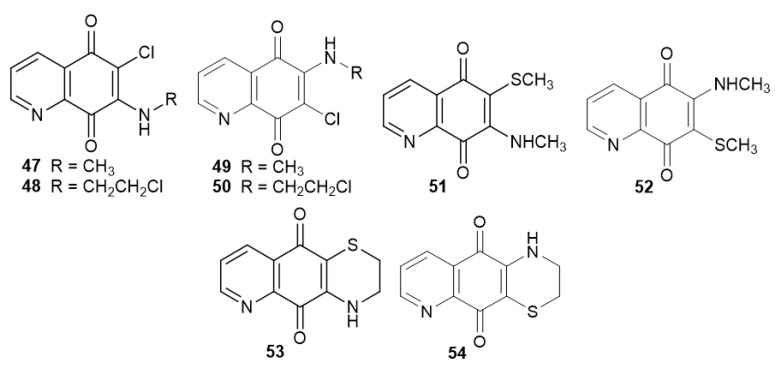
Structure of alkylamino-5,8-quinolinediones **47**–**50** and their modifications **51**–**54**.

Streptonigrin **1** has been tested in vitro and in vivo studies as an anti-leukemia drug, however due to its toxicity, these studies were terminated [15,67]. For this reason, alkylamine derivatives **47**–**48** and **49**–**50** (Figure 8) were evaluated against promyelocytic leukemia (HL60) and T-cells [26].

The regioisomers slightly differed in their activity against HL60 and T-cells, and the IC_50_ values of compounds **47**–**48** and **49**–**50** (Figure 8) were in the range of 1.62–4.30 µM and 2.27–5.21 µM, respectively. The derivatives containing a 2-chloroethylamine group (**48** and **50**) exhibited higher activity against both cancer cell lines than those with a methylamine group (**47** and **49**). Compounds **47** and **49** were used to synthesize derivatives **51** and **52**. The introduction of a thiomethyl group at the C-6 or C-7 position of the 5,8-quinolinedione moiety (**51** and **52**) caused a decrease in the activity against the tested cell lines. Cyclization of **48** and **50** led to the formation of **53** and **54**. A comparison of the activity of compounds **48**, **50**, **53**, and **54** showed that insertion of a 1,4-thiazynyl ring had an adverse impact on the anticancer activity [26].

**Figure 9:**
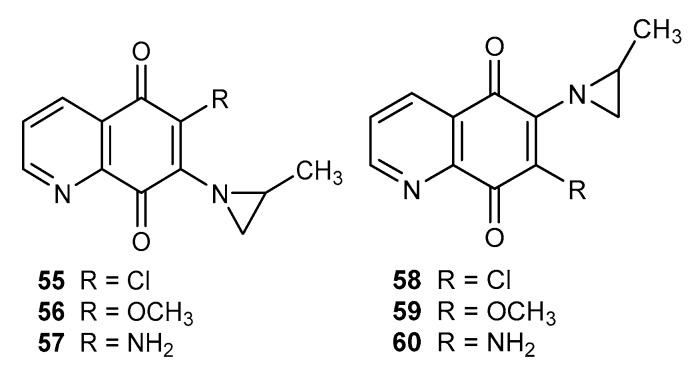
Structure of 2-methylaziridinyl-5,8-quinolinediones **55**–**60**.

Mitomycin C, porfiromycin, carzinophilin, and imexon are natural anticancer agents containing the aziridin moiety [68,69,70,71]. Connection of 5,8-quinolinedione with a 2-methylaziridinyl fragment led to compounds **55**–**57** and **58**–**60** (Figure 9), which exhibited a moderate activity depending on the R group. The research showed that the substituent type affects cytotoxic activity as follows: amine (**57** and **60**) > methoxy (**56** and **59**) > chloride (**55** and **58**) [72].

A comparison of activities in both series of compounds showed that the derivatives containing the aziridinyl moiety at the C-6 position (**58**–**60**) were characterized by a higher cytotoxicity against the SK-OV-3, SK-MEL-2, and XF498 cell lines than the C-7 analogues **55**–**57** [72].

In the literature data, compounds containing an alkoxy, alkynyloxy, or enediyne substituent at the C-7 or/and C-6 position were described. Derivatives of this type seem to be potential anticancer agents [28,51,52].

**Figure 10:**
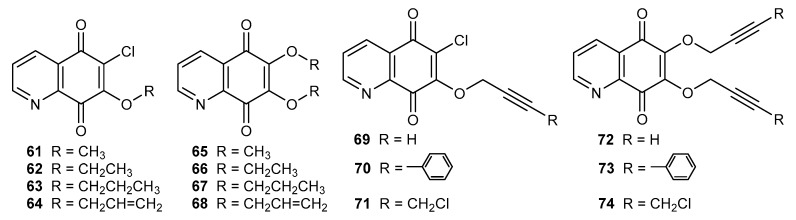
Structure of alkoxy-5,8-quinolinediones **61**–**74**.

The anticancer activity of compounds **61**–**64** and **65**–**68** (Figure 10) was tested by using melanoma (C-32), glioblastoma (SNB-19), and breast cancer (MDA-MB-231) cell lines. The obtained derivatives exhibited a higher activity against all tested cell lines than 6,7-dichloro-5,8-quinolinedione **8**. A comparison of the activity of **61**–**63** and **65**–**67** showed that the introduction of an alkoxy group at the C-6 position led to an increase in the activity against MDA-MB-231 cells. For the SNB-19 line, the activity of mono- **61**–**63** and dialkoxy **65**–**67** derivatives increased together with the elongation of the alkoxy chain. Normal fibroblast (HFF-1) cells were used to evaluate the toxicity of the target derivatives. Compounds containing a propoxy (**63** and **67**) and propenoxy (**64** and **68**) group did not show toxic effects in the tested concentration range [52].

Compounds **69**–**74** (Figure 10) containing substituents with a triple carbon-carbon bond were tested in relation to their anticancer activity against C-32, SNB-19, MDA-MB231, and MCF-7 cell lines. Alkynyloxy derivatives **69**–**71** and **72**–**74** exhibited a higher activity against C-32, SNB-19, and MCF-7 cells than cisplatin and 6,7-dichloro-5,8-quinolinedione **8**. The activity of compounds **69**–**71** and **72**–**74** against C-32 cells depended on the substituent type and the order was as follows: 2-propynoxy (**69** and **72**) > 3-phenyl-2-propynyloxy (**70** and **73**) > 4-chloro-2-butynyloxy (**71** and **74**). The alkynyloxy group at the C-6 position (**72**–**74**) was an important structural element and caused an increase in the activity against the melanoma (C-32) cell line. Mono- and dialkynyloxy compounds (**69**–**71** and **72**–**74**) possessed different activity against breast cancer cell lines and were more cytotoxic for MCF-7 than the MDA-MB-231 cell line. The highest activity against the MCF-7 line was shown by derivative **71**, for which the IC_50_ was equal to 0.04 µM. Additionally, the disubstituted compounds **72**–**74** had a higher selectivity index than monosubstituted derivatives **69**–**71**. Molecular docking study of an NQO1 protein showed that the 5,8-quinolinedione moiety interacted with the hydrophobic active site of the enzyme by binding with Trp105, Tyr128, and Phe178. However, the disubstituted derivatives **72**–**74** created more stable complexes than monosubstituted compounds **69**–**71** [51].

The activity of compounds **63**, **64**, and **69** (Figure 10) against the C-32 cell line increased in the following order: propoxy (**63**) > 2-propenoxy (**64**) > 2-propynoxy (**69**) derivative (Figure 10). For disubstituted compounds (**65**, **68**, and **72**), no correlation between the type of bond in the substituent and activity was observed [28,51,52].

**Figure 11:**
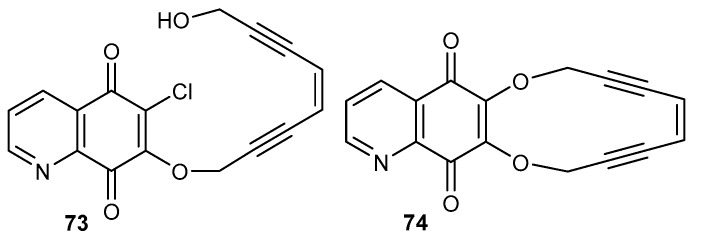
Structure of enediyne derivatives of 5,8-quinolinedione **73**–**74**.

Natural enediyne antibiotics contain a nine or ten-membered unsaturated ring composed of two triple bonds connected through a double bond. Their mechanism of action is based on the transformation of the unsaturated moiety into a 1,4-benzenoid diradical, which can interact with DNA [73,74,75]. Compounds 73 (acyclic) and 74 (cyclic) (Figure 11), obtained as a result of condensation of enediyne-1,8-diol with 6,7-dichloro-5,8-quinolinedione **8,** have been tested as cytotoxic agents on C-32, SNB-19, and MDA-MB-231 cell lines [28].

Compound **74** exhibited a cytotoxic activity against MDA-MB-231 4-times higher than **73**. The acyclic derivative **73** had a higher cytotoxicity than **74** against C-32 and SNB-19, for which the IC_50_ was equal to 3.1 µM and 2.4 µM, respectively. Additionally, acyclic compound **73** did not show any cytotoxic activity against normal fibroblast (HFF-1) cells [28].

**Figure 12:**
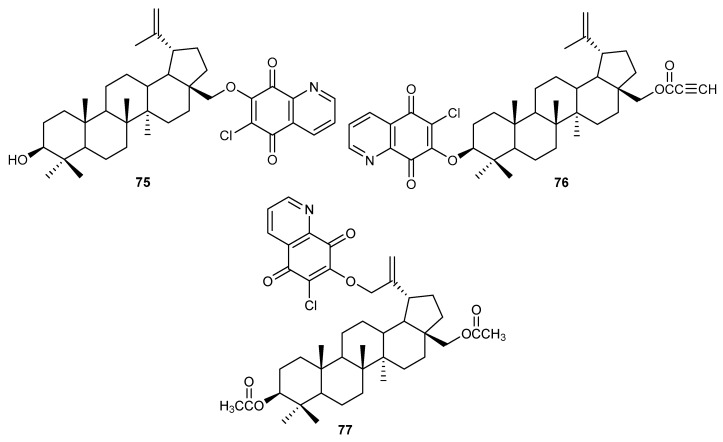
Structure of hybrids betulin-5,8-quinolinedione **75**–**77**.

Betulin was one of the first chemically pure compounds isolated from plant material. Betulin and its semi-synthetic derivatives are characterized by high biological activity including anticancer, antimicrobial, and antiviral activities [76,77,78]. The 5,8-quinolinedione moiety was connected with betulin derivatives to obtain hybrids **75**–**77** (Figure 12). The compounds were tested for their anticancer activity against glioblastoma (SNB-19), melanoma (C-32 and Colo-829), breast (MCF-7, T47D and MDA-MB-231), and lung (A549) cancer cell lines [79].

Hybrids **75**–**77** had different activities against the SNB-19, C-32, MDA-MB-231, and A549 cells lines. The cytotoxic effect was dependent on the location of the 6-chloro-5,8-quinolinedione moiety and the order was as follows: 28-propynoyl-3-betulinyloxy (**76**) > 3,28-diacetyl-30-betulinyloxy (**77**) > 28-betulinyloxy (**75**). However, compounds **75**–**77** exhibited the highest activity against the A549 cell line; the IC_50_ values were in the range 0.45–8.58 µM. An apoptosis assay showed that compound **76** caused a significant increase in TP53 gene expression in A549 and MCF-7 cells. The TP53 gene encodes the p53 protein, which can affect expression of proapoptotic (Bax) and antiapoptotic (Bcl-2) protein [80,81,82]. Hybrid **76** induced a significant expression of Bax and did not affect the expression of Bcl-2 and the ratio of Bax and Bcl-2. The obtained results suggested that the cytotoxic effect of derivative **76** is associated with the mitochondrial apoptosis pathway in the A549 and MCF-7 cell lines. Molecular docking study showed that hybrid **76** was bonded to the NQO1 active site by hydrophobic interaction between the enzyme and the 5,8-quinolinedione moiety [79].

**Figure 13:**
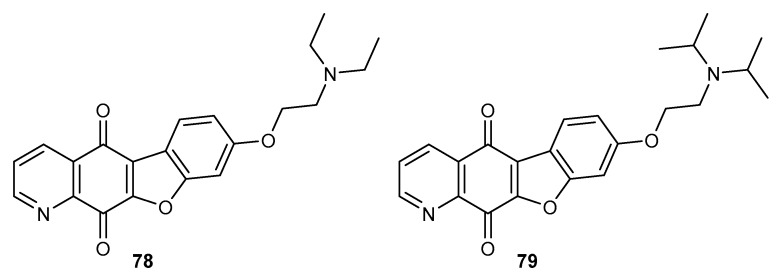
Structure of benzofuro-5,8-quinolinediones **78**–**79**.

Condensation of 6,7-dichloro-5,8-quinolinedione **8** with phenolic derivatives affords benzofuro-5,8-quinolinedione compounds **78**–**79** (Figure 13). These compounds were tested for cytotoxic activity against lung (A549), stomach (SNU-638), colon (HCT116), ovarian (SK-OV-3), and melanoma (SK-MEL-2) cell lines [83].

The kind of substituent at the nitrogen atom in derivatives **78**–**79** affected their anticancer activity. Compound **78**, which contained an ethyl group, exhibited a higher activity against all tested cell lines. In addition, this derivative had activity that was 3-times and 10-times higher against A549 and HCT116 cells than that of doxorubicin, respectively [84]. Most chemotherapeutic agents interact or inhibit topoisomerase I and II, which are involved in the DNA replication process [84]. Compounds **78**–**79** at a 5 µM concentration strongly inhibited the activity of Topo II, and did not interact with Topo I [83].

### 3.2. Antimicrobial Activity

Infections caused by antibiotic-resistant bacteria are one of the most serious problems of modern medicine. The EU and the European Economic Area estimates that in 2015, the median number of infections was 671,689 and more than 33,000 people died of infection [85]. Only one antibiotic, daptomycin, belonging to lipopeptide antibiotics, has been discovered and registered as a drug in the past 50 years [86]. The search for new antibiotics is therefore a particularly important research problem.

Many compounds containing the 1,4-quinone moiety are being investigated for their antibacterial and antifungal properties [87,88,89]. One of the groups that has exhibited promising antibacterial and antifungal activity was constituted by the 5,8-quinolinedione compounds containing the arylamine group [90].

**Figure 14:**
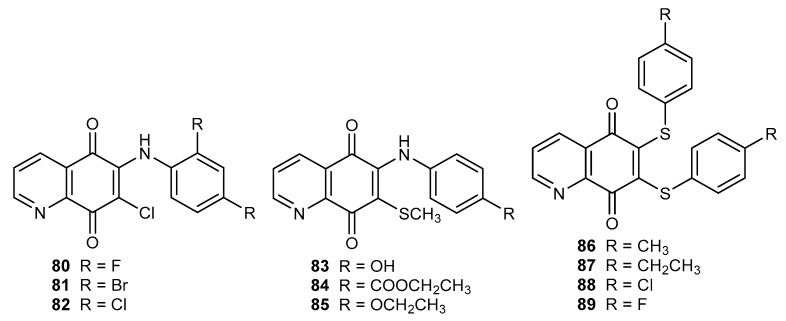
Structure of arylamine (**80**–**82**) and thiol (**83**–**85** and **86**–**89**) derivatives of 5,8-qinolinedione.

The tested compounds **80**–**82** (Figure 14) did not exhibit any activity against Gram-negative bacteria like *E. coli* and *P. aeruginosa*. Activity of derivatives **80**–**82** against Gram-positive bacteria were higher or comparable with that of ampicillin, and depended on the R substituent. The research showed that the R group affects the activity against *MRS aureus*, and this effect decreases in the following order: bromine (**81**) > chloride (**82**) > fluorine (**80**). The arylamine compounds **80**–**82** had a potent antifungal activity against *C. albicans*, *C. albicans L.*, and *A. niger*. Their activities were higher than those of fluconazole and griseofulvin, respectively [90].

Continuing research on antifungal substances, Ryu et al. synthesized two groups of compounds containing a sulfur atom: **83**–**85** with a thiomethyl moiety at the C-7 position and **86**–**89** with 6,7-bisarylthiol substituents (Figure 14) [53,91]. Derivatives **83**–**85** exhibited a potent activity against *Candidia* species and minimum inhibitory concentration (MIC) was within the range of 3.2–25 µg/mL. Conversion of the hydroxy group (**83**) to the ethoxy group (**85**) led to a decrease in the activity against *Candidia krusei* [91]. Bisarylthiol compounds **86**–**89** exhibited a lower activity against *Candida albicans* than flucytosine. The antifungal potency against *Candida krusei* depended on the R group and was as follows: ethyl (**87**) > methyl (**86**) > chloride (**88**) > fluorine (**89**) [53]. A comparison of the activity of derivatives **80**–**89** showed that bisarylthiol **86**–**89** exhibited higher antifungal activity [53,90,91].

**Figure 15:**
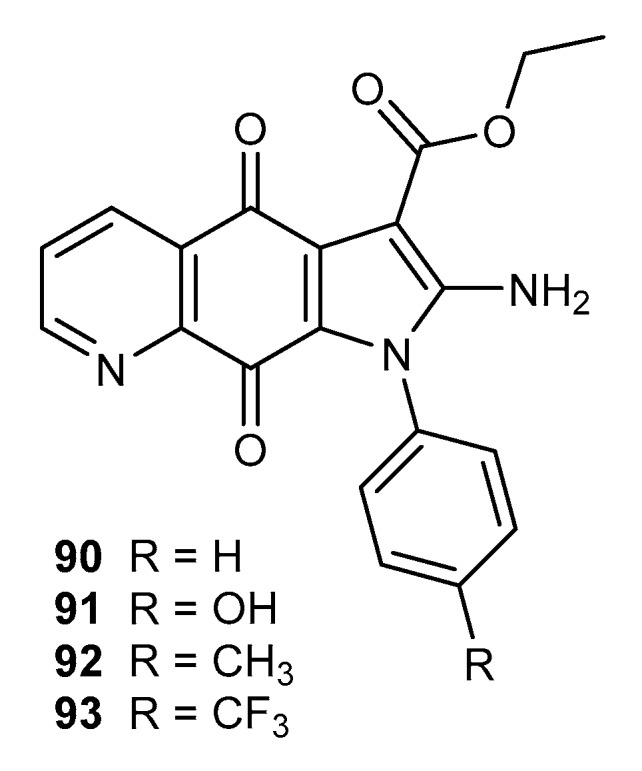
Structure of dihydropyrrole derivatives of 5,8-quinolinedione **90–93**.

Hybrids of the 5,8-quinolinedione moiety with dihydropyrrolyl fragment **90**–**93** (Figure 15) were tested for antifungal activity against the *Candida* species [92].

Compounds **90**–**93** exhibited the highest antifungal activity against *Candida tropicalis* and the MIC was within the range of 0.6–6.3 µg/mL. For these species, the order of activities was as follows: **91** > **93** > **92** > **90** [92].

**Figure 16:**
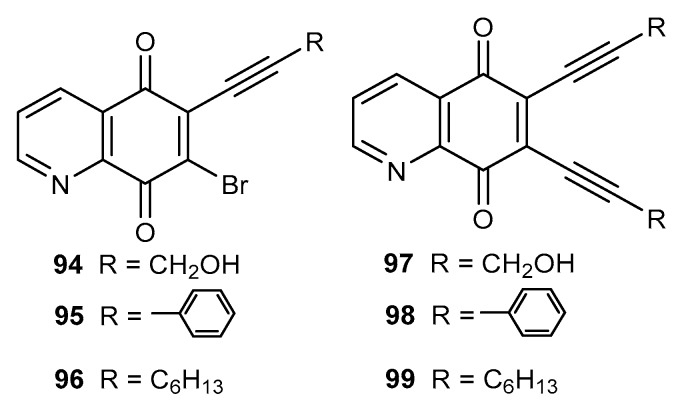
Structure of hybrids alkyne-5,8-quinolinedione **94**–**99**.

Natural enediyne antibiotics are characterized by a high antibacterial activity against Gram-positive and Gram-negative strains [73,74]. The 5,8-quinolinedione moiety contains a double bond between the C-6 and C-7 carbon atom, which enables the enediyne fragment to be obtained by a reaction with terminal alkynes. Ezeokonkwa et al. described the synthesis of mono- (**94**–**96**) (Figure 16) and disubstituent (**97**–**99**) (Figure 16) hybrids of 5,8-quinolinedione with terminal alkynes and their antibacterial activity against *E. coli 12*, *K. pneumonia*, *S. aureus*, and *P. aeruiginosa* [93,94].

Compounds **94**–**96** and **97**–**99** exhibited higher activities than the reference substances, ampicillin and gentamicin. In the series of monoalkynyl derivatives **94**–**96**, potent antibacterial activity against *E. coli*, *K. Pneumonia*, and *P. aeruiginosa* was shown by **94** with a MIC within the range of 0.16–0.32 mg/mL. Replacement of the bromine atom with an alkyne group influenced the antibacterial activity. Unfortunately, the correlation between structure and antibacterial activity of **94**–**96** and **97**–**99** was not observed. In the enediyne compounds **97**–**99**, the strongest activity against *E. coli* and *K. pneumonia* was shown by 6,7-bis(phenylethynyl)-5,8-quinolinedione **98**.

Gram-negative bacteria possess a specific outer membrane, lipid A, as a major component. The LpxC enzyme is responsible for the synthesis of lipid A and is a promising target for the preparation of selective anti-Gram-negative antibiotics [95,96]. The molecular docking study showed that compounds **94**–**99** could bind to LpxC and the obtained ΔG was higher than that of the co-crystalized inhibitor. However, dialkyne derivatives **97**–**99** interacted slightly less with the active site of enzyme [95,96].

The most deadly bacterial disease is tuberculosis, which caused the death of 1.3 million people in 2017. People infected by the HIV virus suffer from the active form of tuberculosis 20–30 times more often than people without HIV [97]. Tuberculosis in HIV-negative people is caused by *Mycobacterium tuberculosis*, but in HIV-positive patients, the etiological factor of the disease may also be *Mycobacterium bovis*, *Mycobacterium africanum*, *Mycobacterium canetti*, and *Mycobacterium microti* [98].

**Figure 17:**
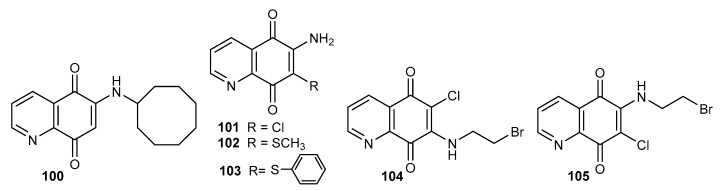
Structure of aliphatic amine derivatives of 5,8-quinolinedione **100**–**105**.

One of the first compounds containing the 5,8-quinolinedione moiety that exhibited activity against *M. tuberculosis* was compound **100** (Figure 17) with a MIC equal to 3.5 µM. Mulchin et al. synthesized a series of amine and thiol derivatives and tested these compounds regarding their anticancer and tuberculostatic activities [26,40].

In the series of compounds **47**–**50** (Figure 8) and **101**–**105** (Figure 17), the highest activity against *M. bovis* was shown by 6-amino-7-chloro-5,8-quinolinedione **101**. Introduction of a thiomethyl or thiophenyl group at the C-7 position decreases the activity. Derivatives containing an amine group at the C-6 position (**50**, **105**) were characterized by higher activity against *M. bovis* than 7-aminosubstituted compounds (**48**, **104**). A comparison of the activities of 2-chloroethylamine (**48** and **50**) (Figure 8) and 2-bromoethylamine (**104**–**105**) (Figure 17) compounds showed that replacing the chloride atom with a bromine atom increased the tuberculostatic activity [26].

**Figure 18:**
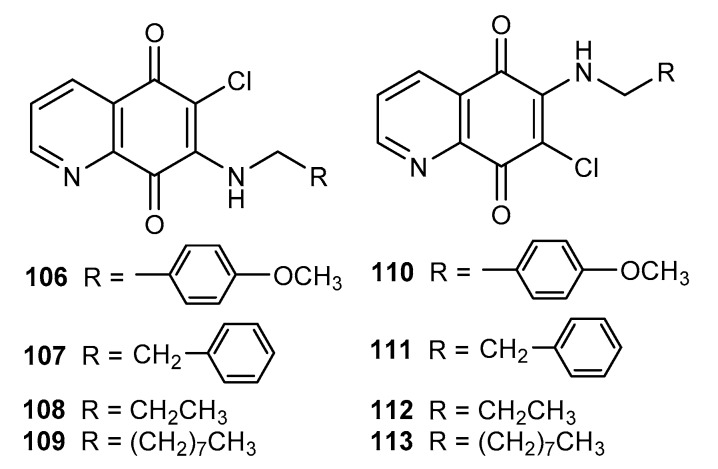
Structure of alkylamino-5,8-quinolinediones **106**–**113**.

Tuberculostatic activities of 6- **106**–**109** and 7-aminosubstituted **110**–**113** 5,8-quinolinediones (Figure 18) were tested against *M. tuberculosis* [40].

Analyzing the obtained results, a general trend was observed. The research proved that the type of substituent and its position had an influence on the activity. A comparison of activities of compounds **106**–**107** and **110**–**111** showed that the derivatives containing a phenyl moiety at the C-7 position (**106** and **107**) had higher activity than the 6-substituted **110**–**111**. An inverse relationship was observed for alkylamine derivatives, and the MIC values for **112**–**113** were lower than those for **108**–**109**. The highest activity against *M. tuberculosis* was exhibited by derivative **45** (Figure 7) with a MIC equal to 8 µM [40].

**Figure 19:**
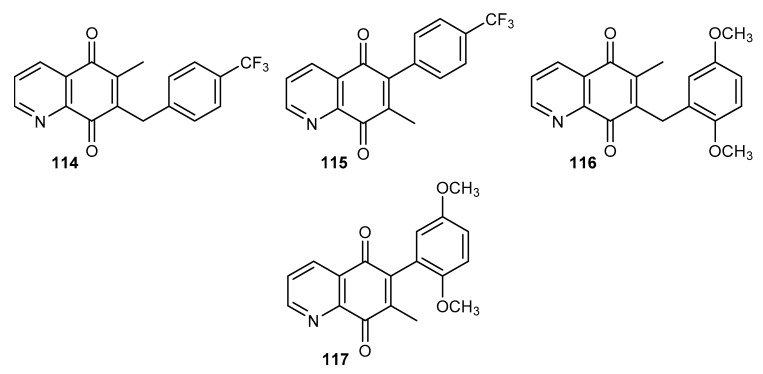
Structure of methyl derivatives of 5,8-quinolinedione **114**–**117**.

The most important problems in Sub-Saharan Africa include malaria caused by the *Plasmodium* parasite. Quinine isolated from cinchona trees was the first anti-malarial drug and is the scaffold for obtaining more effective substances [99,100]. Lanfranchi et al. synthetized a series of derivatives that were tested on two types of *P. falciparum* (Figure 19) [101].

Compounds **115** and **117** (Figure 19) containing a phenyl substituent at the C-6 position did not exhibit any activity against chloroquine-sensitive 3D7 and multi-resistance Dd2 strains of *P. falciparum*. Derivative **114** showed a higher activity against the 3D7 strain with an IC_50_ equal to 727 nM. Compound **116** exhibited a comparable activity against both strains of *P. falciparum* [101].

**Figure 20:**
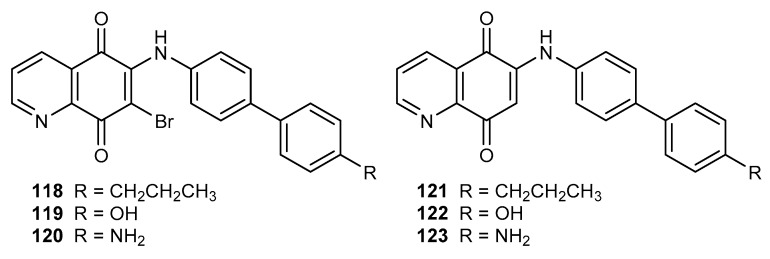
Structure of biphenyl-4-amino-5,8-quinolinediones **118–123**.

The 6-substituted derivatives of 7-bromo-5,8-quinolinedione (**118**–**120**) and 5,8-quinolinedione (**121**–**123**) (Figure 20) were tested on a chloroquine-sensitive strain of *P. falciparum* (NF54) [102].

In the series of tested compounds, only **118** showed antiplasmodial activity (IC_50_ equal to 3 µM). Comparing the activities of 5,8-quinoinediones **118** and **122**, it was found that the bromine atom at the C-7 position was necessary to maintain the antimalarial activity [102].

### 3.3. Anti-HIV Activity

**Figure 21:**
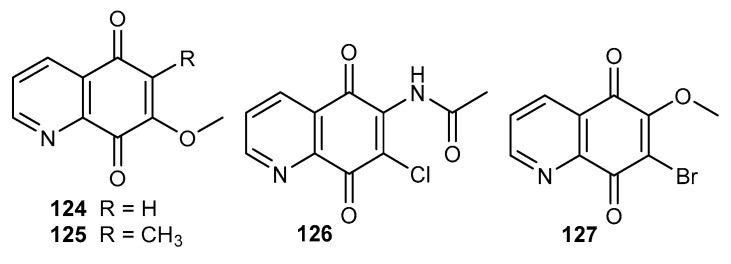
Structure of disubstituted derivatives of 5,8-quinolinedione **124**–**127**.

Human immunodeficiency virus (HIV), which causes acquired immune deficiency syndrome (AIDS), is one of the most serious health problems in the worldwide. It is estimated that in 2018, 37.9 million people lived with the HIV virus and 5000 people are infected by this virus every day [103]. Streptonigrin **1** and its derivatives inhibit HIV reverse transcriptase (RT) and the postulated mechanism of action assumes an interaction of the quinone moiety with the active site of this enzyme [104]. Derivatives **124**–**125** at a concentration 10 µg/mL caused over 80% inhibition of HIV reverse transcriptase activity (Figure 21). The activity of 7-methoxy-5,8-quinolinedione **124** was slightly higher. At the same concentration, derivatives **124**–**125** did not affect cellular DNA polymerases, which could suggest a non-toxic effect of these compounds [104].

Alfadhli et al. tested compounds **8** and **126**–**127** as inhibitors of RNA binding to the HIV-1 matrix (MA) protein (Figure 21) [105]. All tested derivatives inhibited MA-RNA binding and the percentage of RNA binding inhibition was equal to 99–73%. Moreover, **8** and **127** caused the crosslinking of cytosine-containing proteins, MA and PrGap proteins in the HIV-1 virus. Unfortunately, the compounds were characterized by a high toxicity against the human T-cell line [105].

The 5,8-quinolinedione compounds were also tested as anti-inflammatory agents [26], inhibitors of enzymes such as nitric synthase, cyclooxygenase-2 [106], acetylcholine induced vasorelaxation of rat aorta with the endothelium [107,108], tyrosine phosphatase [109], and picornavirus 3C protease [110].

## 4. Conclusions

The 5,8-quinolinedione scaffold occurs in many natural and synthetic compounds. In recent years, interest in its potential therapeutic properties against many diseases has increased. A comparison of activities of the 6- and 7-substituted compounds showed that it depended on the type of substituent and the substitution site in the 5,8-quinolinedione moiety. According to this review, in most cases, the 6-substituted derivatives had higher activities, but is not a rule. 

Despite the large number of natural and synthetic active derivatives, only a few of them have qualified for clinical trials [7,8,9,10]. The most important problem in this class of compounds is due to their toxicity. For this reason, further research in all areas of the presented biological activity of 5,8-quinolinedione compounds is still needed to design novel, more effective, and less toxic structures.

The discovery of novel modifications of the 5,8-quinolinedione moiety at C-6 and C-7 positions may lead to interesting medical applications in the future.

## Figures and Tables

**Table 1:** Reaction conditions for the preparation of compounds **7**–**9**.

Compound.	Reaction Conditions	Yield [%]	Ref.
**7**	Bis-[(trifluoroacetoxy)iodo]benzene, water-acetonitrile, 0 ℃	82–89	[31,32]
	oxygen, methanol-dichloromethane, 15 ℃	64	[33]
	Potassium dihydrogenphosphate, potassium nitrososulfonate, methanol, room temperature	92	[34]
	phenyliodine(III) diacetate, water-acetonitrile, 0 ℃	86	[35]
	5,10,15,20-Tetrakisphenylporphyrin, oxygen, dichloromethane, 20 ℃	82	[36]
	[Bis(acetoxy)iodo]benzene, water-acetonitrile, 0 ℃	82	[37]
**8**	Hydrogenchloride, sodium chlorate, 30–60 ℃	17–30	[26,30,38,39,40]
	Hydrogenchloride, nitric acid, 90 ℃	39	[41]
	Multi-step reaction with 3 steps: 1: hydrogenchloride, sodium nitrite, water, 0–4 °C 2: sodium dithionite, sulfuric acid, sodium hydroxide 3: hydrogenchloride, sodium chlorate	No date	[42,43]
**9**	Multi-step reaction with 3 steps: 1: hydrogenchloride, sodium nitrite, water, 0–4 °C 2: sodium dithionite, sulfuric acid, sodium hydroxide 3: sodium bromate, hydrogen bromide	No date	[42,44]
